# Primary small intestinal extranodal NK/T cell lymphoma, nasal type with kidney involvement: a rare case report and literature review

**DOI:** 10.1186/s13000-022-01254-z

**Published:** 2022-10-05

**Authors:** Shuyan Mao, Changying Diao, Lei Cao

**Affiliations:** 1 Department of Pathology, Xuzhou Central Hospital, No.199, Jiefang South Road, 221009 Xuzhou, Jiangsu China; 2 Department of Pathology, Shanghai Pudong New Area People’s Hospital, No.490, Chuanhuan South Road, Chuansha town, 200120 Shanghai, China

**Keywords:** NK/T cell lymphoma, EN-NK/T-NT, ENKTL, Small intestine, Kidney, EBV

## Abstract

**Background:**

Extranodal NK/T cell lymphoma, nasal type (EN-NK/T-NT) is a rare and aggressive type of non-Hodgkin’s lymphoma. EN-NK/T-NT seldom occurs in the gastrointestinal tract, and renal involvement is relatively rare.

**Case presentation:**

Here we report a case of primary small intestinal EN-NK/T-NT with kidney involvement. We present the case of a 71-year-old female who was admitted to our hospital for coronary heart disease with a fever of unknown origin. Laboratory examination showed renal impairment and PET/CT showed a locally thickened wall of the small intestine, abnormally increased FDG metabolism in the right lower abdomen, and multiple slightly high-density masses with abnormal increased FDG metabolism in the right kidney. The gross specimen showed a grayish-white lump located in the ileum approximately 15 cm away from the ileocecum, and two grayish-white lumps located in the upper and lower poles of the right kidney, respectively. The pathological diagnosis was EN-NK/T-NT. The patient died approximately 10 months after the operation.

**Conclusion:**

EN-NK/T-NT is a rare type of non-Hodgkin’s lymphoma and may develop insidiously, with fever as the only clinical manifestation. The disease was found to be difficult to diagnose in the early stage, resulting in a highly aggressive clinical course and short survival time.

## Background

Extranodal NK/T cell lymphoma (ENKTL) is a specific non-Hodgkin’s lymphoma, which is relatively rare in Western countries; however, it is the most common type of peripheral T-cell lymphoma in China. ENKTL mainly occurs extranodally, most commonly in the nasal cavity and nasopharynx, and seldom in the soft tissue, skin, gastrointestinal tract, and testis. ENKTL is generally believed to originate from NK cells or cytotoxic T cells [1] and is associated with Epstein-Barr virus (EBV) infection [2]. ENKTL also presents with complex molecular genetic changes and an aggressive clinical course.

Case presentation

Summary of case.

The patient was a 71-year-old woman with a history of hypertension, type 2 diabetes, and coronary heart disease. In recent months, she presented with chest distress in the precardiac region after activity, with recurrent attacks that were accompanied by tachypnea and hypodynamia. She presented with edema of both lower limbs over 1 day and was admitted to the hospital, where she had a fever of unknown origin after admission. The patient had abdominal distention, and no obvious nasal congestion, nasal hemorrhage, abdominal pain, and diarrhea symptoms.

Laboratory examination showed creatinine, urea and urinary microalbumin concentrations of 205.7 µmol/L, 17.2µmol/L, and 594 mg/L, respectively. In the urine, the red blood cell count was 20.6/µl and the white blood cell count was 211.0/µl. Other relevant examinations showed no obvious abnormalities.

Computed tomography (CT) showed a locally thickened wall of the small intestine in the right lower abdomen, blurred fat space around the lesions, and a nodular shadow in the right kidney.

**Fig. 1:**
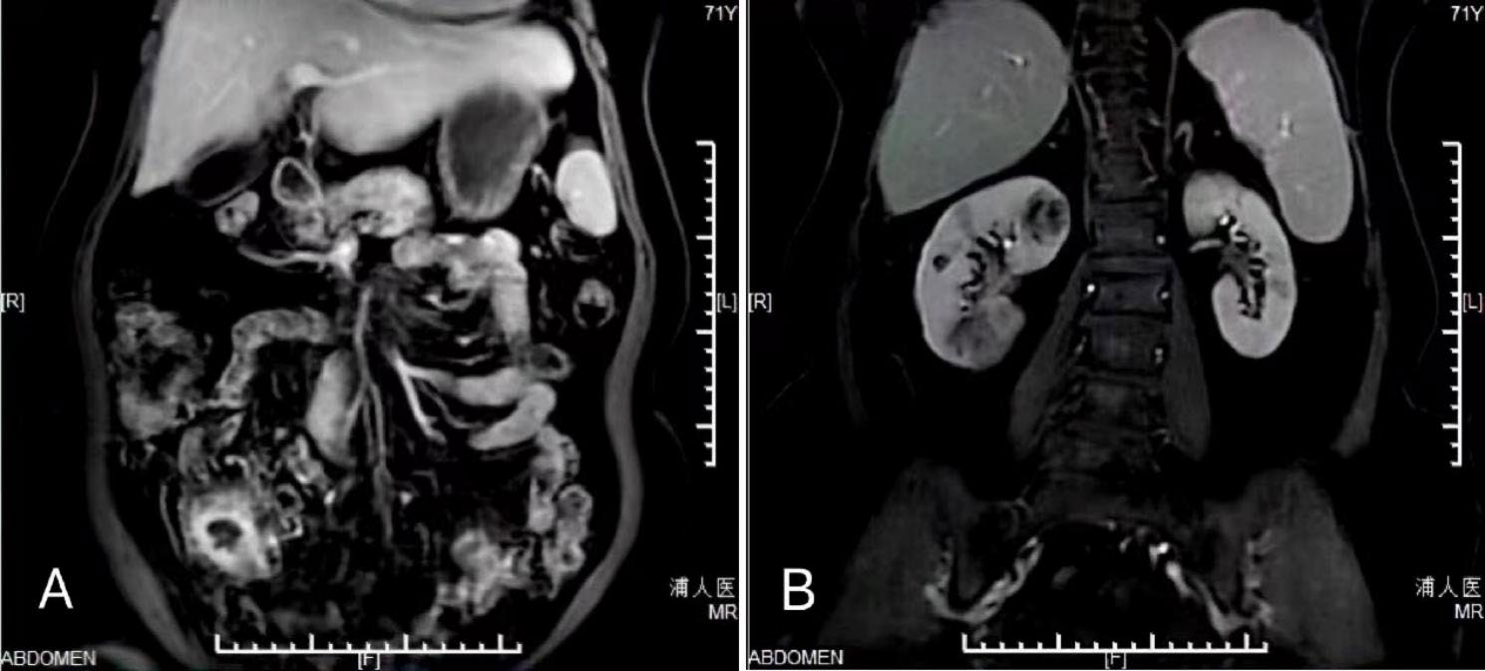
Magnetic resonance imaging show that the local small intestinal wall is irregularly thickened and intensifies in the right lower abdomen **(A)**, with multiple hypersignal intensities observed in the right kidney **(B)**

Magnetic resonance imaging (MRI) showed an irregularly thickened local small intestine wall, which was intensified in the right lower abdomen (Fig. 1 A), and multiple hypersignal intensities in the right kidney (Fig. 1B), suggesting space-occupying lesions.

Positron emission tomography/computed tomography (PET/CT) showed multiple slightly high-density masses with abnormally increased fluorodeoxyglucose (FDG) metabolism in the right kidney, as well as a locally thickened small intestinal wall with abnormally increased FDG metabolism in the right lower abdomen. Malignant lesions should be considered.

The patient was treated with anti-hypertensive, hypoglycemic, oxygen, and anti-infection therapy, and then transferred to the surgical ward for operation. Intraoperative observation showed a mass was located in the ileum approximately 15 cm away from the ileocecal, infiltrated into the serosal membrane of the intestinal wall, and adhered to the ileum 35 cm away from the ileocecal, ulceration on the surface of the mass. The right kidney was cut open along the renal hilum, two grayish-white masses located in the upper and lower poles of the right kidney, respectively.

Pathological findings.

Grossly, a grayish-white mass, 2.8 × 1.6 × 1.5 cm in size, was located in the ileum approximately 15 cm away from the ileocecum. The mass showed surface ulceration formation, involving the full thickness of the ileum wall. One grayish-white mass was located in the upper pole of the right kidney, approximately 3.5 × 3.5 × 3.0 cm in size, which was well circumscribed with adjacent tissue. Another grayish-white mass was located in the lower pole of the right kidney, approximately 2.5 × 2.0 × 1.0 cm in size, and was formed as a well-circumscribed spheroid adjacent to the renal capsule.

**Fig. 2:**
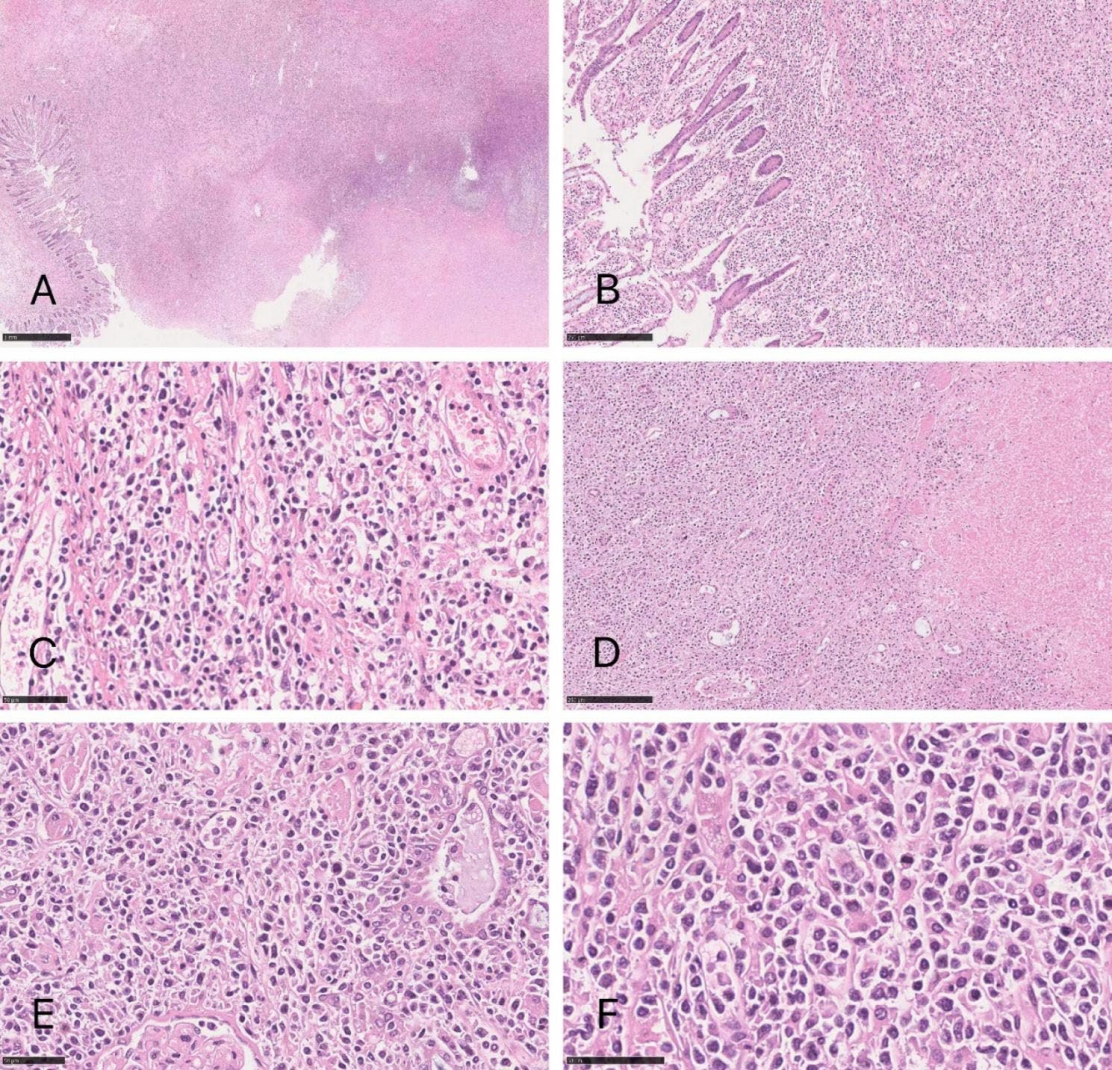
The tumor cells are diffusely distributed, infiltrating the entire intestinal wall **(A, ×20; B, ×100)** and renal parenchyma with obvious coagulation necrosis in some areas **(D, ×100)**. The tumor cells are mainly of small to medium size, with blood vessel invasion observed **(C, ×400; E, ×400; F, ×600)**

Microscopically, the tumor cells were diffusely distributed, infiltrating the full-thickness of the intestinal wall (Fig. 2 A, 2B) and renal parenchyma, with obvious coagulation necrosis in some areas (Fig. 2D). The tumor cells were diverse in morphology, mainly small to medium-size cells, mixed with a few large cells. The nuclear morphology of the tumor cells was irregular with hyperchromatic nuclei, only sporadic small nucleoli, and visible nuclear fragmentation. The tumor cells invaded the blood vessels, and necrosis was found in the vascular wall (Fig. 2 C, 2E, 2 F). A few reactive inflammatory cells were observed in the surrounding area of the tumor.

**Fig. 3:**
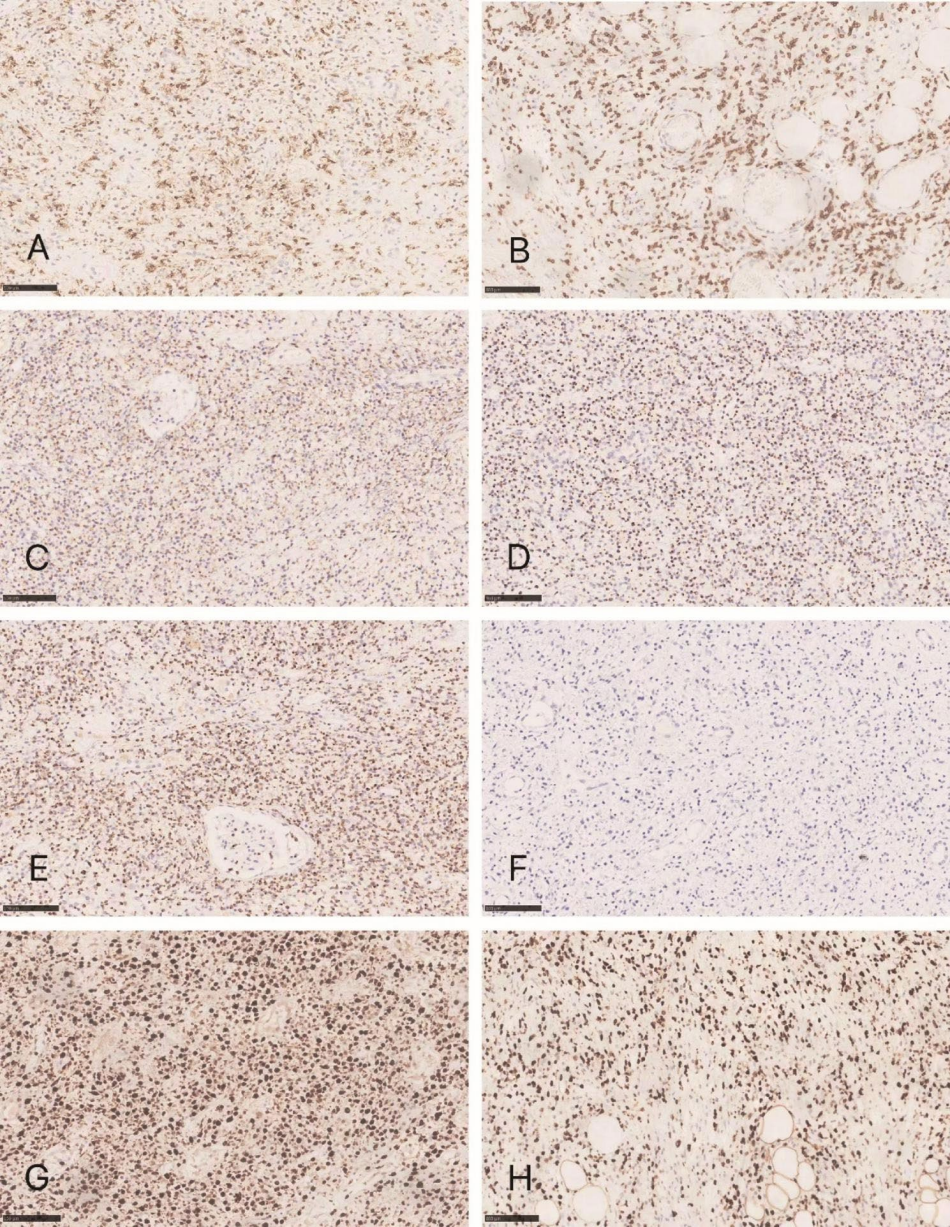
Immunohistochemical analysis show that tumor cells are positive for CD3 **(A)**, CD56 **(B)**, granzyme B **(C)**, TIA-1 **(D)**, and perforin **(E)** and negative for CD20 **(F)**. The Ki-67 proliferation index reach up to 90% (Fig. 3G). In situ hybridization for EBER show strong positivity in most of the tumor cells (Fig. 3 H).

Immunohistochemically, the staining results of tumor cells in the small intestine and kidney were consistent; the tumor cells were positive for CD3 **(**Fig. 3 A**)**, CD56 **(**Fig. 3B**)**, granzyme B **(**Fig. 3 C**)**, TIA-1 **(**Fig. 3D**)**, and perforin **(**Fig. 3E**)**, while negative for CD4, CD8, CD5, Desmin, CD34, CKpan, CD20 **(**Fig. 3 F**)**, CD30, SMA, CD79ɑ, and PAX8. The Ki-67 proliferation index reached up to 90% **(**Fig. 3G**)**. In situ hybridization for EBER showed strong positivity in most of the tumor cells **(**Fig. 3 H**)**. And Molecular analysis for T cell receptor (TCR) gene rearrangement showed there was no clonal rearrangement. Based on morphological features, immunohistochemical results, and molecular data, the case was diagnosed as extranodal NK/T cell lymphoma, nasal type (EN-NK/T-NT).

**Treatment and outcome**.

After two treatment courses of gemcitabine and oxaliplatin, the patient was discontinued due to intolerance and switched to toripalimab immunotherapy, followed by withdrawal due to lung infection, and improved infection after anti-inflammatory and hormone therapy. The patient developed intermittent low-grade fever, was generally unwell, and died approximately ten months after operation.

**Discussion and Conclusions**.

ENKTL mainly involves the nasal and nasopharynx, with clinical features including a stuffy nose and nasal hemorrhage, as tumors grow along the respiratory tract and spread to the paranasal sinus and palate. ENKTL seldom involves the skin, gastrointestinal tract, and testis, but there are reports of ENKTL involving the blood vessels, uterus, and adrenal gland [3–5]. Renal involvement is rarely reported. Lymph node involvement occurs in approximately 20% of cases [6], although primary lymph node involvement is rare. Disseminated ENKTL may involve multiple organs and present with the leukemia phase [7].

According to the cell of origin, ENKTL may be classified into T and NK cell subtypes, TCR genes are clonally rearranged in the T cell subtype but not in the NK cell subtype [1, 8]. Both express the cytotoxic proteins GrB, TIA-1, and Perforin. ENKTL may express CD20 infrequently; although co-expression of CD20 in T-cell lymphoma has been reported previously, the TCR rearrangement of these cases was negative, suggesting an NK cell origin. Nevertheless, the significance of CD20 expression remains unclear [9, 10].

An increasing number of studies have suggested that EBV plays an important role in the pathogenesis of ENKTL. EBV is a human gamma herpes double-stranded DNA virus that mainly infects B cells, as well as T cells, NK cells, and epithelial cells [11]. The EBV infection pattern includes proliferative infection and latent infection. At present, ENKTL is considered latency I or II, which corresponds to different expressions of Epstein-Barr Nuclear Antigen (EBNA) and latent membrane protein (LMP). EBV-encoded small RNA (EBER) in situ hybridization has shown that almost all ENKTL tumor cells are positive [12, 13]. Thus, it is important to diagnose ENKTL by detecting the expression of EBER.

ENKTL presents with complex molecular genetic changes, with deletion of 6Q21 being the most common chromosome change. The tumor suppressor genes associated with this change are PRDM1, PTPRK, HACE1, and FOXO3 [14]. Deletion, methylation or mutation of PRDM1 cause inactivation, resulting in changes in NK cell homeostasis [15, 16].

In collaboration with 22 clinical centers, Professor Zhao conducted a multiomics study on 128 samples of NK/T cell lymphoma and proposed three molecular subtypes: TSIM, MB and HEA, corresponding to different EBV patterns, genomic changes, and clinical outcomes [17]. EBV gene transcription varies among the three molecular subtypes: The TISM subtype is associated with the expression of BALF3, an EBV lytic gene, which enhances genomic instability and regulates TP53 target genes, leading to malignant transformation; the MB subtype shows a low expression of EBV genes, especially LMP1, suggesting that this subtype is latent infection type I; and the HEA subtype is characterized by enhanced expression of BNFR1, a lytic gene, which interacts with DAX3X to promote viral latency and cell immortalization [17]. Three molecular subtypes are related to the origin of tumor cells and prognosis. TISM subtypes express more NK cell genes, with the activation of the JSK-STAT signaling pathway, and overexpress TP53 and PD-L1. Overexpression of PD-L1/2 may enhance the therapeutic effect of PD1 blockade [18]; thus, PD-L1/2 may be a potential therapeutic target for TISM subtypes. The MB subtype is closely related to MYC overexpression, with mutations of the tumor suppressor gene MGA and activation of the MAPK, NOTCH, and WNT signaling pathways [19].The HEA subtype is an epigenetic alteration, mainly involving abnormal acetylation of histone, which activates the NF-KB and TCR signaling pathways. This subtype expresses more T-cell genes [20]. Among the three subtypes, the MB subtype has the worst prognosis.

The gastrointestinal tract is the most common site of extranodal lymphoma, with susceptibility factors including infection, celiac disease, and inflammatory bowel disease [21, 22]. The most common type of gastrointestinal tract primary T cell lymphoma is enteropathy-associated T cell lymphoma (EATL), divided into types 1 and 2. Most patients with type 1 present with a history of celiac disease and chronic malabsorption. Additionally, the tumor cells are medium to large cells, expressing T cell and cytotoxic markers, negative EBER by in situ hybridization, and with the distinct nucleolus. Type 2 is called monomorphic epitheliotropic intestinal T-cell lymphoma (MEITL), is not associated with celiac disease, and has obvious epitheliotropic characteristics. The tumor cells are monomorphic and small to medium in size. Additionally, an inflammatory background is relatively rare, with obvious increases in intraepithelial lymphocytes, atrophy of the adjacent intestinal villus, expression of T cell and cytotoxic markers, TCR gene rearrangement positive, and generally negative for EBER by in situ hybridization [23–25]. As a differential diagnosis, intestinal inert T/NK lymphoproliferative disease should be considered, and proliferative small lymphocytes are paramorphic, with non-destructive infiltration, generally low Ki67 expression, and EBER negative.

Primary renal lymphoma is a non-Hodgkin’s lymphoma restricted to the kidney without systemic disease, Primary renal lymphoma often occurs in the unilateral kidney, or shows bilateral involvement, invading the adjacent tissue, including the perirenal fat, psoas major, pancreas, and small intestine [26]. Primary renal lymphoma is rare; when extranodal lymphoma presents as kidney involvement, it is regarded as secondary renal lymphoma [27]. ENKTL includes nasal and non-nasal. Nasal involvement is more common than non-nasal. In the diagnosis of extra-nasal tumors, the primary nasal with extra-nasal invasion should be excluded [28]. In this case, The patient had no definite symptoms of nasal involvement, and PET/CT also showed no space-occupying lesion in the nasopharynx. Therefore, we diagnosed it as primary small intestinal extranodal NK/T cell lymphoma, nasal type with kidney involvement.

Patients with EN-NK/T-NT have different prognoses. The overall 5-year survival rate of patients with limited lesions and good response to treatment is better, while most patients with tumor metastases have an overall survival rate of less than 1 year. Poor prognostic factors include high stage, high international prognostic index, bone involvement, and high levels of circulating EBV DNA [29, 30]. The detection of circulating EBV DNA can be used to monitor the disease [31].

We reported a rare case of primary small intestinal EN-NK/T-NT with kidney involvement. The patient had no obvious abdominal pain and diarrhea symptoms; the tumor was found on examination and had metastasized.

We concluded that ENKTL may develop insidiously, with fever as the only clinical manifestation. The disease was found to be difficult to diagnose in the early stage, resulting in a highly aggressive clinical course and short survival. With continuous in-depth study of ENKTL, several potential therapeutic targets have been identified, which could provide new clues for targeted therapy and improve prognosis.

## Data Availability

All data and materials generated or analyzed during this study are included in this article.

## Abbreviations

CT: computed tomography.
FDG: fluorodeoxyglucose.
MRI: magnetic resonance imaging.
PET/CT: positron emission tomography/computed tomography.
ENKTL: extranodal NK/T cell lymphoma.
EBV: Epstein-Barr virus.
EBER: EBV-encoded small RNA.
EN-NK/T-NT: extranodal NK/T cell lymphoma, nasal type.
EBNA: Epstein-Barr Nuclear Antigen.
LMP: latent membrane protein.
EATL: enteropathy-associated T cell lymphoma.
MEITL: monomorphic epitheliotropic intestinal T-cell lymphoma.
TCR: T cell receptor.

## References

[1] Stewart CA, Walzer T, Robbins SH, Malissen B, Vivier E, Prinz I (2007). Germ-line and rearranged Tcrd transcription distinguish bona fide NK cells and NK-like gammadelta T cells. Eur J Immunol.

[2] Tse E, Kwong YL (2013). How I treat NK/T-cell lymphomas. Blood.

[3] Wei J, Wu H, Sun M, Liu W, Meng L (2012). Primary endometrial natural killer (NK)/T cell lymphoma: case report and review of literature. Eur J Gynaecol Oncol.

[4] Yan J, Zhang F, Luo D, Yao S, Chen Y, Xu F (2017). Intravascular NK/T-cell lymphoma: a series of four cases. Int J Clin Exp Pathol.

[5] Ichikawa S, Saito K, Fukuhara N, Yokoyama H, Onodera K, Onishi Y (2020). Primary adrenal extranodal NK/T-cell lymphoma: A case report and literature review. Leuk Res Rep.

[6] Barrionuevo C, Zaharia M, Martinez MT, Taxa L, Misad O, Moscol A (2007). Extranodal NK/T-cell lymphoma, nasal type: study of clinicopathologic and prognosis factors in a series of 78 cases from Peru. Appl Immunohistochem Mol Morphol.

[7] Tse E, Kwong YL (2019). NK/T-cell lymphomas. Best Pract Res Clin Haematol.

[8] Pongpruttipan T, Sukpanichnant S, Assanasen T, Wannakrairot P, Boonsakan P, Kanoksil W (2012). Extranodal NK/T-cell lymphoma, nasal type, includes cases of natural killer cell and alphabeta, gammadelta, and alphabeta/gammadelta T-cell origin: a comprehensive clinicopathologic and phenotypic study. Am J Surg Pathol.

[9] Huang Y, Chen S, Wei R, Guo X, Yang X, Cao Q (2020). CD20-positive extranodal NK/T cell lymphoma: clinicopathologic and prognostic features. Virchows Arch.

[10] Yang S, Ba Y, Jiang G (2021).

[11] Lv H, Ye L, Liu Q, Li SG, Li T, Huang NL (2019). S-S-PEG-COOH Self-Assembled Monolayer on Gold Surface Enabled a Combined Assay for Serological EBV Antibody Isotypes. Proteom Clin Appl.

[12] Houldcroft CJ, Kellam P (2015). Host genetics of Epstein-Barr virus infection, latency and disease. Rev Med Virol.

[13] Okuno Y, Murata T, Sato Y, Muramatsu H, Ito Y, Watanabe T (2019). Defective Epstein-Barr virus in chronic active infection and haematological malignancy. Nat Microbiol.

[14] Huang Y, de Reynies A, de Leval L, Ghazi B, Martin-Garcia N, Travert M (2010). Gene expression profiling identifies emerging oncogenic pathways operating in extranodal NK/T-cell lymphoma, nasal type. Blood.

[15] de Mel S, Hue SS, Jeyasekharan AD, Chng WJ, Ng SB (2019). Molecular pathogenic pathways in extranodal NK/T cell lymphoma. J Hematol Oncol.

[16] Zhang Z, Liang L, Li D, Nong L, Liu J, Qu L (2017). Hypermethylation of PRDM1/Blimp-1 promoter in extranodal NK/T-cell lymphoma, nasal type: an evidence of predominant role in its downregulation. Hematol Oncol.

[17] Xiong J, Cui BW, Wang N, Dai YT, Zhang H, Wang CF (2020). Genomic and Transcriptomic Characterization of Natural Killer T Cell Lymphoma. Cancer Cell.

[18] Kwong YL, Chan TSY, Tan D, Kim SJ, Poon LM, Mow B (2017). PD1 blockade with pembrolizumab is highly effective in relapsed or refractory NK/T-cell lymphoma failing l-asparaginase. Blood.

[19] Li X, Cheng Y, Zhang M, Yan J, Li L, Fu X (2018). Activity of pembrolizumab in relapsed/refractory NK/T-cell lymphoma. J Hematol Oncol.

[20] Calao M, Burny A, Quivy V, Dekoninck A, Van Lint C (2008). A pervasive role of histone acetyltransferases and deacetylases in an NF-kappaB-signaling code. Trends Biochem Sci.

[21] Alvarez-Lesmes J, Chapman JR, Cassidy D, Zhou Y, Garcia-Buitrago M, Montgomery EA (2021). Gastrointestinal Tract Lymphomas. Arch Pathol Lab Med.

[22] Peng JC, Zhong L, Ran ZH (2015). Primary lymphomas in the gastrointestinal tract. J Dig Dis.

[23] Al Somali Z, Hamadani M, Kharfan-Dabaja M, Sureda A, El Fakih R, Aljurf M (2021). Enteropathy-Associated T cell Lymphoma. Curr Hematol Malig Rep.

[24] Jiao G, Zheng Z, Jiang K, Zhang J, Wang B (2014). Enteropathy-associated T-cell lymphoma presenting with gastrointestinal tract symptoms: A report of two cases and review of diagnostic challenges and clinicopathological correlation. Oncol Lett.

[25] Chen C, Gong Y, Yang Y, Xia Q, Rao Q, Shao Y (2021). Clinicopathological and molecular genomic features of monomorphic epitheliotropic intestinal T-cell lymphoma in the Chinese population: a study of 20 cases. Diagn Pathol.

[26] Bokhari SRA, Inayat F, Bokhari MR, Mansoor A. Primary renal lymphoma: a comprehensive review of the pathophysiology, clinical presentation, imaging features, management and prognosis. BMJ Case Rep. 2020;13(6).10.1136/bcr-2020-235076. 10.1136/bcr-2020-235076 PMC7304849 32554453

[27] Ganeshan D, Iyer R, Devine C, Bhosale P, Paulson E (2013). Imaging of primary and secondary renal lymphoma. AJR Am J Roentgenol.

[28] Tse E, Au-Yeung R, Kwong YL (2019). Recent advances in the diagnosis and treatment of natural killer/T-cell lymphomas. Expert Rev Hematol.

[29] Chim CS, Ma SY, Au WY, Choy C, Lie AK, Liang R (2004). Primary nasal natural killer cell lymphoma: long-term treatment outcome and relationship with the International Prognostic Index. Blood.

[30] Cheung MM, Chan JK, Lau WH, Ngan RK, Foo WW (2002). Early stage nasal NK/T-cell lymphoma: clinical outcome, prognostic factors, and the effect of treatment modality. Int J Radiat Oncol Biol Phys.

[31] Au WY, Pang A, Choy C, Chim CS, Kwong YL (2004). Quantification of circulating Epstein-Barr virus (EBV) DNA in the diagnosis and monitoring of natural killer cell and EBV-positive lymphomas in immunocompetent patients. Blood.

